# Alcohol and Tobacco Use in a Tuberculosis Treatment Cohort during South Africa’s COVID-19 Sales Bans: A Case Series

**DOI:** 10.3390/ijerph18105449

**Published:** 2021-05-19

**Authors:** Bronwyn Myers, Tara Carney, Jennifer Rooney, Samantha Malatesta, Laura F. White, Charles D. H. Parry, Tara C. Bouton, Elizabeth J. Ragan, Charles Robert Horsburgh, Robin M. Warren, Karen R. Jacobson

**Affiliations:** 1 Alcohol, Tobacco and Other Drug Research Unit, South African Medical Research Council, Tygerberg 7505, South Africa; tcarney@mrc.ac.za (T.C.); cparry@mrc.ac.za (C.D.H.P.); 2 Division of Addiction Psychiatry, Department of Psychiatry & Mental Health, University of Cape Town, Cape Town 7701, South Africa; 3 Section of Infectious Diseases, Department of Medicine, Boston Medical Center, Boston, MA 02119, USA; jennifer.rooney@bmc.org (J.R.); tcbouton@bu.edu (T.C.B.); elizabeth.ragan@bmc.org (E.J.R.); rhorsbu@bu.edu (C.R.H.J.); kjacobso@bu.edu (K.R.J.); 4 Department of Biostatistics, School of Public Health, Boston University, MA 02119, USA; samalate@bu.edu (S.M.); lfwhite@bu.edu (L.F.W.); 5 Department of Psychiatry, Stellenbosch University, Tygerberg 7505, South Africa; 6 Departments of Epidemiology, Biostatistics and Global Health, Boston University School of Public Health, Boston, MA 02119, USA; 7 Department of Science and Innovation, National Research Foundation Centre of Excellence in Biomedical Tuberculosis Research, South Africa Medical Research Council for Tuberculosis Research, Stellenbosch University, Tygerberg 7505, South Africa; rw1@sun.ac.za

**Keywords:** COVID, alcohol sales ban, South Africa, heavy drinking, alcohol policy

## Abstract

Background: South Africa temporarily banned alcohol and tobacco sales for about 20 weeks during the COVID-19 lockdown. We described changes in alcohol and tobacco consumption after implementation of these restrictions among a small number of participants in a tuberculosis treatment cohort. Method: The timeline follow-back procedure and Fägerstrom test for nicotine dependence was used to collect monthly alcohol and tobacco use information. We report changes in heavy drinking days (HDD), average amount of absolute alcohol (AA) consumed per drinking day, and cigarettes smoked daily during the alcohol and tobacco ban compared to use prior to the ban. Results: Of the 61 participants for whom we have pre-ban and within-ban alcohol use information, 17 (27.9%) reported within-ban alcohol use. On average, participants reported one less HDD per fortnight (interquartile range (IQR): −4, 1), but their amount of AA consumed increased by 37.4 g per drinking occasion (IQR: −65.9 g, 71.0 g). Of 53 participants who reported pre-ban tobacco use, 17 (32.1%) stopped smoking during the ban. The number of participants smoking >10 cigarettes per day decreased from 8 to 1. Conclusions: From these observations, we hypothesize that policies restricting alcohol and tobacco availability seem to enable some individuals to reduce their consumption. However, these appear to have little effect on the volume of AA consumed among individuals with more harmful patterns of drinking in the absence of additional behavior change interventions.

## 1. Introduction

During the COVID-19 pandemic, several countries restricted the sale and distribution of alcohol, including India, Thailand, and South Africa [1,2,3]. Decisions to restrict alcohol sales were based on evidence that limiting availability reduces levels of alcohol consumption and associated harms [4]. These harms include the potential impact of drinking on immunological responses to COVID-19, adherence to preventative measures when under alcohol’s influence, and the impact of alcohol-attributable harms on health system capacity to manage increased demand during COVID-19 surges [5]. Countries have differed in the severity of these restrictions, with South Africa implementing some of the most restrictive measures and for longer periods of time [2,6].

South Africa entered a national lockdown on 27 March 2020 in which only essential items were available for purchase. Alcohol and tobacco were listed as non-essential, meaning a total ban on the sale and distribution of these substances. These lockdown bans were implemented with little forewarning, restricting bulk purchasing and stockpiling of these substances. Decision-makers aimed to lower alcohol-related emergency care visits and hospitalizations, increasing health system capacity for increasing COVID-19 cases [6]. Reports that smoking increased risk of severe COVID-19 disease and the need for intensive care and ventilation underpinned the decision to ban tobacco [7]. The alcohol sales ban was lifted on 1 June but reinstated on 13 July until 17 August 2020.

Describing whether and how individual alcohol use changed is important for monitoring policy impact and for identifying unintended and unexpected consequences of these public health policy changes [8]. For instance, recorded alcohol and tobacco sales were lower during these bans [2], suggesting that many individuals without an alcohol use disorder (AUD) reduced their consumption. However, as seen in other parts of the world [9], individuals with heavier and more harmful patterns of alcohol use may have struggled to stop drinking particularly given corresponding COVID-19 restrictions on access to the country’s scarce substance use treatment services [10,11]. Such individuals may have resorted to using illicitly obtained or undocumented alcohol and tobacco to avoid forced abstinence and corresponding symptoms of withdrawal. This is feasible given that undocumented alcohol (including traditional home-brewed alcohol and other non-commercial beverages) is readily available in all communities [12] and accounts for approximately a quarter of all alcohol consumption in South Africa [13]. Furthermore, illicit trade in tobacco (either from cross-border smuggling or illicit production) to evade tax and reduce price, is well-established in this context [14].

Yet, no published research currently exists on the effects of these bans on individual consumption patterns with any level of detail or granularity. This is partly because population-level data on alcohol consumption is not readily available for this purpose. Although other high-income countries used electronic cross-sectional surveys to assess alcohol consumption during COVID-19 [15,16], this is less feasible in South Africa where problematic patterns of alcohol use are concentrated in population subgroups of poorer socio-economic status with fewer resources to participate in online surveys. Further, information on drinking patterns prior to these alcohol bans (that could serve as a baseline for assessing the impact of alcohol policy changes) is not readily available. In this setting, alcohol consumption data are not routinely collected from individuals seeking health care or the general population and when collected rarely includes detailed information on volume of alcohol consumed [17].

Given this lack of data, we sought to leverage an existing longitudinal patient cohort to allow for the generation of hypotheses about the effects of these bans on individual alcohol and tobacco use. More specifically, this report aims to describe observed changes to established patterns of alcohol and tobacco consumption among a case series of participants in a tuberculosis (TB) treatment cohort study during the course of South Africa’s COVID-19 related alcohol and tobacco bans.

## 2. Methods

We present data from the Tuberculosis Treatment and Alcohol Use Study (TRUST; ClinicalTrials.gov Identifier NCT02840877). Participants were ≥15 years old and initiating treatment for active pulmonary TB in Worcester, a rural town in the Western Cape. Participants consented to providing information on their alcohol and tobacco use at baseline; monthly for the six months of TB treatment; and at three, six, nine, and 12-month post-treatment endpoints. Recruitment and study processes are described elsewhere [18].

Socio-demographic information (age, sex) was collected at baseline. Risk of clinically significant depression was assessed at baseline with the Center for Epidemiologic Studies Depression (CES-D) scale, where scores ≥ 16 are indicative of significant symptoms of depression. [19] Pre-ban, Alcohol Use Disorders Identification Test (AUDIT) scores were used to classify participants as having low risk of alcohol-related harms (scores < 8), hazardous or harmful use (scores of 8–20), and possible alcohol use disorder (AUD) if scores ≥ 20 [20,21]. We defined excessive alcohol use as AUDIT scores ≥ 8. At baseline and each monthly follow-up, we collected information on daily quantity and type of alcohol consumed over a two-week period using the timeline follow back (TLFB) procedure [22]. TLFB was used to define the average amount of absolute alcohol (AA) consumed per drinking occasion and the number of heavy drinking days (HDD) in the two weeks prior to the interview. Study nurses were trained to probe participants about their use of all types of alcohol, including informally produced alcohol (such as home-brews) and alcohol sold in non-commercial packaging. To aid recall and help standardize the quantity of alcohol consumed, participants were shown pictures of frequently consumed formal and informally produced alcoholic beverages in this setting in containers of various sizes.

In keeping with the International Alcohol Control Study [23], heavy drinking was defined as consuming ≥96 g of absolute alcohol (AA, about eight standard drinks) for men and ≥72 g AA (about six standard drinks) for women. Standard drinks in South Africa contain approximately 12 g AA. During the COVID-19 ban, we added additional questions to these standard monthly assessments that examined any current alcohol use that was not limited to the two-week window for the TLFB assessment (with response options being yes or no) and perceived changes in access to alcohol as a result of the COVID-19 restrictions (with response options being no effect on access, less access during the ban, and more access during the ban).

At baseline, and at monthly assessments during the ban, we assessed current tobacco use and number of cigarettes smoked per day with the Fagerström Test for Nicotine Dependence [24]. Participants were prompted to report any tobacco use, whether from a legal source or an illegal source. The within-ban monthly follow-up assessments also included a question examining perceived changes in access to tobacco during the COVID-19 sales ban (with response options being no effect on access to cigarettes, less access to cigarettes, and more access to cigarettes).

Using R software, we examined changes in alcohol and tobacco use among participants for whom we have within-ban alcohol TLFB or tobacco information. Among participants who continued using alcohol and tobacco during the COVID-19 ban, we explored changes in the number of HDD, average amount of AA consumed per drinking day, and number of cigarettes smoked per day from their last pre-ban assessment. Heatmaps were constructed to depict change in the average amount of AA consumed per drinking day and the average number of HDD over three timepoints (baseline, last pre-ban monthly assessment, and monthly assessment that occurred during the period of the ban) among participants who reported past two-week alcohol use on the TLFB at any of the pre-ban timepoints.

## 3. Results

**Figure 1:**
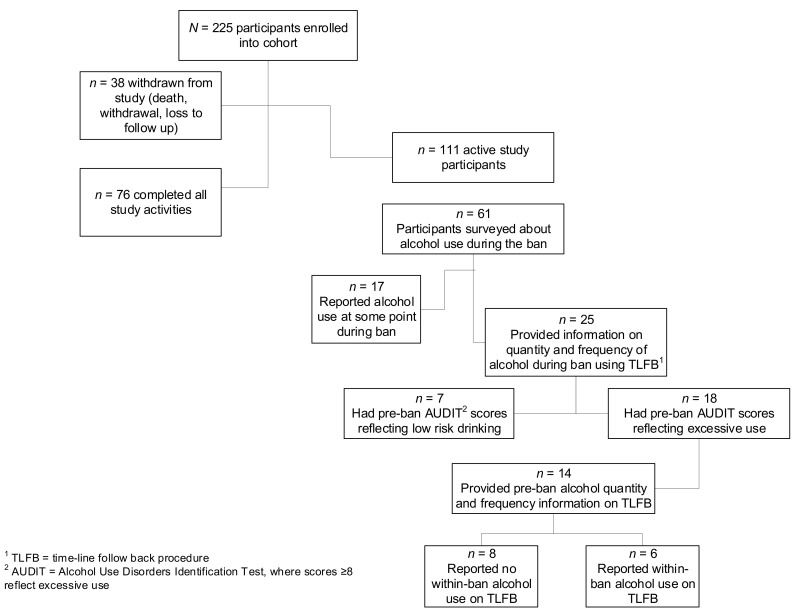
Study flowchart of alcohol reporting during COVID-19 bans.

During the national lockdown, all in-person research activities were temporarily halted from 27 March 2020. By this time, 225 participants had been enrolled of which 76 had completed all study activities as planned and 38 had ended study participation prior to completion for various reasons including death (*n* = 5), voluntary withdrawal (*n* = 6), TB treatment relapse or failure (*n* = 7), moved out of area (*n* = 3), and loss to follow up/no longer contactable (*n* = 17). Of the remaining 111 active participants, 41 were in the six-month treatment phase (and still requiring monthly follow up) and 70 were in the post-treatment follow up phase. The study was permitted to resume follow-up appointments telephonically with the remaining 111 active participants from 22 June 2020, but no new participant enrolments were permitted during this phase of the pandemic (see Figure 1 for study flowchart).

To correspond with the tobacco and alcohol ban periods, analyses are restricted to those participants who completed telephonic follow up assessments as per protocol during the period of the bans and provided information on either tobacco use between 22 June 2020 and 17 August 2020 (*n* = 73 of the 111 participants active in the study), or alcohol use during the period of the second alcohol ban between 13 July 2020 and 17 August 2020 (*n* = 61), or in combination. No follow up assessments were conducted during the period of the first alcohol ban (27 March 2020 to 1 June 2020).

With the exception of assigned sex, the 61 participants who provided within-ban information on alcohol use did not differ significantly from the 225 participants recruited into the TRUST cohort by 1 April 2020 (see Table 1 for comparison of demographic characteristics).

Of these 61 participants, 17 (27.9%) reported some alcohol use during the ban. More than half of these 17 participants (*n* = 9, 52.9%) reported that the ban had little effect on their access to alcohol, although 8 (47.1%) reported less access.

We examined changes in alcohol use among 25 participants who reported alcohol use on the TLFB during their within-ban monthly assessment. These participants were predominantly women (*n* = 15, 60.0%), had a median age of 37 (26–48), with 52% (*n* = 13) being unemployed. Seven (28%) of these participants had AUDIT scores in the low-risk range, 10 (40.0%) had AUDIT scores indicating hazardous or harmful use, and eight (32%) had AUDIT scores suggestive of a possible AUD. Apart from an overrepresentation of women, the socio-demographics of this sample did not significantly differ from those of the full TRUST cohort (*n* = 225).

Eighteen of these 25 participants had pre-ban AUDIT scores indicating excessive use, of whom 14 reported alcohol use on one of the pre-ban TLFB assessments. During the ban, 6 of these 14 participants continued drinking: two with baseline scores indicative of hazardous/harmful drinking and four with a possible AUD (see Figure 1 for more information). Most participants who continued drinking had clinically significant symptoms of depression at baseline (*n* = 5, 83.3%).

**Figure 2:**
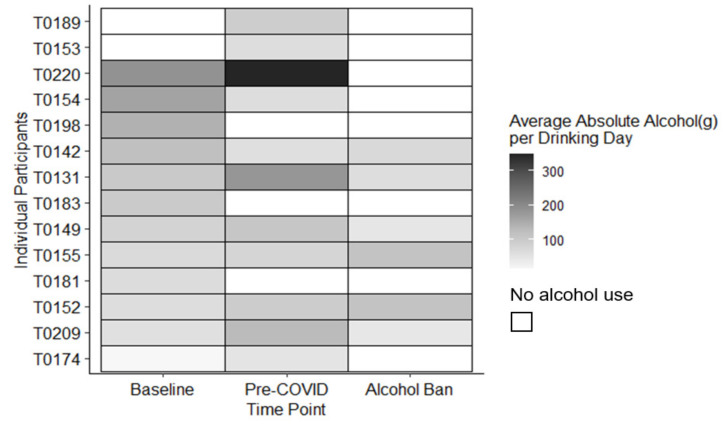
Changes in the average absolute amount of alcohol (grams) consumed per drinking occasion among participants who provided pre-ban and within-ban alcohol use information (*n* = 14).

**Figure 3:**
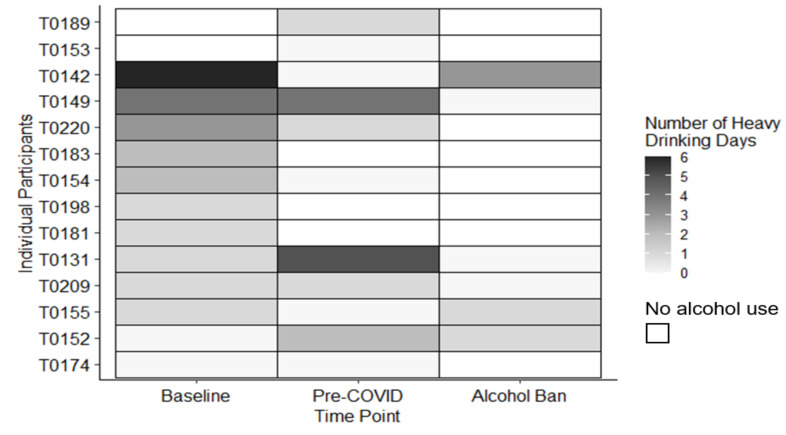
Changes in the number of heavy drinking days among participants who provided pre-ban and within-ban alcohol use information (*n* = 14).

Among the six participants who continued drinking, the median amount of AA consumed increased by 37.4 g per drinking day (interquartile range (IQR): −65.9 g to 71.0 g) during the ban. This increase is roughly equivalent to an additional three units of alcohol per drinking occasion. As reflected in Figure 2, this increase appears largely driven by three participants who substantially increased the amount of AA they consumed per drinking occasion. In contrast, the median number of HDD decreased by one day (IQR: −4 to 1) during the alcohol ban. Figure 3 indicates that only three participants reported any HDD during the ban. Two of these participants reduced the number of HDD between their baseline and pre-COVID study visits but seemed to relapse to previous patterns of drinking during the alcohol ban.

Of the 73 participants who provided data on tobacco use during the ban, 46 (63.0%) reported smoking prior to the ban. Two-thirds (*n* = 31, 68.9%) of these participants reported reduced access to cigarettes during this period, although more than a quarter (*n* = 12, 26.7%) reported that their access remained unchanged. We examined changes in tobacco use among 53 participants who reported pre-ban smoking. Among these participants, 36 (67.9%) continued smoking despite the ban. Prior to the ban, 28 of these 36 participants (77.8%) smoked 10 or fewer cigarettes per day, 6 (16.6%) smoked between 11–20 cigarettes, and 2 smoked more than 20 cigarettes per day (5.6%). During the ban, the proportion of participants smoking more than 10 cigarettes per day decreased from 22.2% (*n* = 8) to 2.8% (*n* = 1) of participants.

## 4. Discussion

Restricting alcohol and tobacco availability is considered an effective policy measure for reducing alcohol- and tobacco-attributable harms, [25] but has been contested by the alcohol and tobacco industries [4]. Local evidence to support the implementation of alcohol supply restrictions has also been sparse. While South Africa has intermittently implemented alcohol and tobacco bans as a temporary strategy for conserving health system capacity during COVID-19 surges, these periods provided an opportunity to preliminary describe changes in individual alcohol consumption behavior among TRUST study participants observed after the implementation of these policy changes.

More than two-thirds of participants reported alcohol cessation and one-third reported tobacco cessation during the period of the ban. However, our observations suggest that these alcohol and tobacco bans did not eliminate alcohol and tobacco use among all individuals. This is consistent with the experiences of other countries that have banned alcohol at various points in history [26,27]. Despite almost half of the study participants surveyed during the period of the tobacco ban and second alcohol ban reporting less access to alcohol and two-thirds reporting less access to cigarettes, almost a third continued drinking and two-thirds continued smoking tobacco during these bans.

In the absence of providing medication-assisted treatment for smoking cessation and other evidence-based smoking cessation aids, it is not surprising that two-thirds of individuals included in this small case series continued to smoke during the tobacco ban. Nonetheless, these participants did reduce the intensity of tobacco use, reportedly smoking fewer cigarettes per day during the period of the tobacco ban. This is a positive step towards reducing the harms associated with continued smoking.

In contrast, when we examined changes in drinking patterns among people with baseline AUDIT scores indicative of heavy drinking, 67% reported no alcohol use during the ban. With heavy drinking being a major contributor to alcohol-attributable harms, this observation provides some initial support for decisions to restrict the availability of formally produced alcohol as a strategy for reducing alcohol-attributable harms. Among the third of participants (mostly with baseline AUDIT scores suggestive of a probable AUD) who continued drinking, we observed shifts in drinking frequency and quantity. On average, participants decreased the number of HDD, with several not reporting any HDD during the ban. Reductions of even one to two HDD per month can decrease the number of alcohol-attributable events [28]. However, even in this small case series, a few participants increased their HDD. As reported in other studies [29], the continued (and increased) use of alcohol noted among these participants may have been a coping response to pandemic-related stress and depression. This is plausible given that participants who continued drinking also reported clinically significant symptoms of depression. Alcohol bans may be better tolerated and humane if access to AUD and mental health services to help people cope is simultaneously improved [30,31].

Although we noted an overall reduction in number of HDD among participants, the average amount of alcohol consumed per drinking occasion appeared to increase by an additional three drinks. It seems that limiting alcohol access may have reduced participants’ opportunities to drink, but some participants appeared to compensate by consuming more when they had the chance. This observation was unexpected, raising concerns about potential unanticipated adverse outcomes of the ban given associations between greater volumes of alcohol consumption and risk of harm [28]. Further, we noted that participants who decreased their alcohol consumption still consumed high volumes of alcohol on drinking days. We hypothesize that restricting the availability of formally produced alcohol in the absence of psychosocial interventions to support behavior change may have little effect on the volume of AA consumed among individuals with more harmful patterns of drinking.

Findings should be interpreted with caution given the small sample size and because the sample is restricted to persons being treated for TB from a single geographic area known for its high levels of alcohol availability and consumption due to local alcohol production. We acknowledge the relationship between socio-economic status, alcohol consumption and risk of TB [32], and therefore the possibility that patterns of alcohol consumption observed in this cohort may differ from the general population. However, the purpose of this report is to describe changes in drinking among participants in the TRUST cohort as a result of this short-term alcohol and tobacco policy change that can be used to generate hypotheses for further testing in rigorously designed and more representative studies evaluating alcohol and tobacco policies. As TRUST was not intended as an evaluation of alcohol and tobacco policy, there are other design limitations that require consideration. Although participants were asked to report any kind of alcohol or tobacco use (regardless of source), we did not collect specific information on how and where participants manage to source alcohol and tobacco during the bans. This information is potentially important for the successful implementation of alcohol and tobacco policies and should be collected by future studies. Including additional data time-points in this report may have provided further insights into how these policy changes affected participants’ behavior. The small sample size and the fact that participants were all at different stages of TB treatment and post-treatment follow up precluded us from including all data time-points in the analyses. We plan to address this limitation once the full cohort has been enrolled and has completed all study activities. In addition, as we only resumed research activities after the first alcohol ban had been lifted, we were unable to explore how alcohol consumption patterns changed during the six-week break in the alcohol sales ban.

Despite these limitations, our observations provide insights into some of the possible effects of restricting alcohol and tobacco availability on individual behavior. We observed that some individuals stopped or reduced their alcohol and tobacco use during these bans, but others with more problematic use continued drinking at harmful levels. Based on these observations, we hypothesize that restricting access to alcohol and tobacco, as a singular policy intervention, may be insufficient for decreasing risk of alcohol and tobacco-attributable harms—at least among populations with high levels of baseline alcohol problems. If this hypothesis is valid, a multipronged policy approach to reducing alcohol and tobacco harms where efforts to restrict availability are accompanied by either the introduction, scale up of alcohol and tobacco-focused behavior change interventions and treatment support, or both, may yield greater population-level impacts. Recently proposed changes in alcohol and tobacco legislation and policy frameworks [2,33] provide the ideal opportunity to fully test this hypothesis.

## Figures and Tables

**Table 1:** Demographic characteristics of participants in the Tuberculosis Treatment and Alcohol Use Study (TRUST) cohort who provided within-ban alcohol data.

	TRUST Participants Not included in the Analyses (*n* = 164)	TRUST Participants included in the Analyses (*n* = 61)	Total *n* = 225	*p* Value
Assigned sex: female	57 (34.8%)	34 (55.7%)	91 (40.4%)	0.004
Median age (Interquartile range)	38 (29, 49)	42 (28, 49)	39 (29, 49)	0.587
Education below 9th grade	69 (42.1%)	26 (42.6%)	95 (42.2%)	0.941
Unemployed	99 (60.4%)	40 (65.6%)	139 (61.8%)	0.475
HIV Positive	51 (31.1%)	16 (26.2%)	67 (29.8%)	0.478
Previous history of TB	63 (38.4%)	25 (41.0%)	88 (39.1%)	0.726
Problem alcohol use	104 (63.4%)	39 (63.9%)	143 (63.6%)	0.943
Tobacco use	113 (68.9%)	43 (70.5%)	156 (69.3%)	0.818
History of imprisonment	112 (68.3%)	44 (72.1%)	156 (69.3%)	0.579

## Data Availability

Data are available on request from Dr. Karen Jacobson.
